# Dysregulated expression of Fau and MELK is associated with poor prognosis in breast cancer

**DOI:** 10.1186/bcr2350

**Published:** 2009-08-11

**Authors:** Mark R Pickard, Andrew R Green, Ian O Ellis, Carlos Caldas, Vanessa L Hedge, Mirna Mourtada-Maarabouni, Gwyn T Williams

**Affiliations:** 1 Institute for Science and Technology in Medicine and School of Life Sciences, Keele University, Huxley Building, Keele ST5 5BG, UK; 2 Division of Pathology, School of Molecular Medical Sciences, University of Nottingham and Nottingham University Hospitals, Derby Road, Nottingham NG7 2UH, UK; 3 Cancer Research UK Cambridge Research Institute and Department of Oncology, University of Cambridge, Hills Road, Cambridge CB2 0RE, UK

## Abstract

**Introduction:**

Programmed cell death through apoptosis plays an essential role in the hormone-regulated physiological turnover of mammary tissue. Failure of this active gene-dependent process is central both to the development of breast cancer and to the appearance of the therapy-resistant cancer cells that produce clinical relapse. Functional expression cloning in two independent laboratories has identified Finkel–Biskis–Reilly murine sarcoma virus-associated ubiquitously expressed gene (*Fau*) as a novel apoptosis regulator and candidate tumour suppressor. Fau modifies apoptosis-controller Bcl-G, which is also a key target for candidate oncoprotein maternal embryonic leucine zipper kinase (MELK).

**Methods:**

We have used RNA interference to downregulate *Fau *and *Bcl-G *expression, both simultaneously and independently, in breast cancer cells *in vitro *to determine the importance of their roles in apoptosis. Expression of *Fau*, *Bcl-G *and *MELK *was measured by quantitative RT-PCR in breast cancer tissue and in matched breast epithelial tissue from the same patients. Expression data of these genes obtained using microarrays from a separate group of patients were related to patient survival in Kaplan–Meier analyses.

**Results:**

siRNA-mediated downregulation of either *Fau *or *Bcl-G *expression inhibited apoptosis, and the inhibition produced by combining the two siRNAs was consistent with control of Bcl-G by Fau. Fau expression is significantly reduced in breast cancer tissue and this reduction is associated with poor patient survival, as predicted for a candidate breast cancer tumour suppressor. In addition, *MELK *expression is increased in breast cancer tissue and this increase is also associated with poor patient survival, as predicted for a candidate oncogene. *Bcl-G *expression is reduced in breast cancer tissue but decreased *Bcl-G *expression showed no correlation with survival, indicating that the most important factors controlling Bcl-G activity are post-translational modification (by Fau and MELK) rather than the rate of transcription of Bcl-G itself.

**Conclusions:**

The combination of *in vitro *functional studies with the analysis of gene expression in clinical breast cancer samples indicates that three functionally interconnected genes, *Fau*, *Bcl-G *and *MELK*, are crucially important in breast cancer and identifies them as attractive targets for improvements in breast cancer risk prediction, prognosis and therapy.

## Introduction

Breast cancer is the most common cancer in women in the developed world [1], and is the second leading cause of cancer-related deaths after lung cancer. Despite recent advances in therapy, the development of therapy-resistant breast cancer cells is a major cause of death. Initial or acquired resistance to endocrine therapy or to trastuzumab (Herceptin) is seen in a majority of patients [2,3]. These difficulties provide a powerful incentive for further molecular dissection of the processes involved in breast cancer development and therapy.

Cellular self-destruction through the active gene-dependent process of apoptosis is fundamental to breast epithelial cell physiology. Oestrogen is critical to homeostasis in breast tissue, and high concentrations stimulate cell proliferation and suppress cell death (for example [4]). In healthy breast tissue, lowering of oestrogen concentrations both removes the proliferative stimulus and alleviates the suppression of cell death, resulting in apoptosis. The physiological balance between proliferation and cell death breaks down during the development of breast cancer, and the failure of breast cancer cells to engage the apoptosis programme is crucial for oncogenesis, as is the case for other cancers [5,6].

Induction of apoptosis is also critical to the success of breast cancer therapy. Oestrogen blockade by anti-oestrogens lifts the suppression of apoptosis in oestrogen receptor-positive cells, resulting in the elimination of susceptible cells [7]. Many other anticancer therapies act not by direct destruction of the cancer cell, but by producing intracellular damage to which the cell responds through self-destruction by apoptosis [8,9]. Failure of apoptosis produces drug-resistant cancer cells that can give rise to clinical relapse [10].

The central importance of apoptosis in the development and therapy of breast cancer has stimulated many investigations aimed at improving understanding of the process at the molecular level. Such an understanding is essential to provide the rational basis for targeting the molecules that play critical roles in the control of cell death and survival in order to develop novel and effective therapies. Functional expression cloning provides a powerful and proven strategy for the direct identification of molecules controlling cell death through their effects on cell survival. This strategy has successfully identified many genes that play important roles in controlling the cell fate in both healthy tissue and cancers, and has highlighted important mechanisms controlling cancer cell death that had escaped detection by other methods (for example [11-16]). One gene identified directly through its control of cell death and survival by two independent laboratories is Finkel–Biskis–Reilly murine sarcoma virus-associated ubiquitously expressed gene (*Fau*) [12,15]. The Finkel–Biskis–Reilly murine sarcoma oncogenic virus contains a sequence antisense to *Fau *that increases the tumorigenicity of the virus, suggesting that Fau can act as a tumour suppressor [17]. Fau induces apoptosis in several cell types and is required for T-cell apoptosis induced by DNA-damaging agents such as UV radiation and cisplatin [15]. Serial analysis of gene expression has indicated that *Fau *is downregulated early in breast cancer development [18].

The molecular mechanism of action of Fau involves the transfer of its ubiquitin-like FUBI domain to cellular target proteins as a post-translational modification analogous to other ubiquitin-like modifications, such as SUMO [19]. One prominent target for FUBI modification is Bcl-G (Bcl2L14 [20]), a member of the Bcl-2 family of apoptosis-controlling proteins that frequently plays an important role in cancer development and therapy (reviewed in [6]). This observation [21] immediately suggests a potential mechanism for the control of apoptosis by Fau; that is, through regulation of the effects of Bcl-G.

Further attention has been focused on Bcl-G because of its identification [22] as an important target for maternal embryonic leucine zipper kinase (MELK). MELK is a recently identified protein kinase and candidate oncoprotein that is upregulated in several types of cancer, including breast cancer [22,23], and is associated with resistance to apoptosis [22]. Once again, the modification of Bcl-G (in this case by phosphorylation) provides an attractive mechanism of action for the observed pro-survival effects of MELK [22].

## Materials and methods

### Breast cancer cell culture

The human breast cancer cell line T-47D was maintained in MEM (M5650; Sigma, Gillingham, Dorset, UK) supplemented with 5% heat-inactivated FCS, 2 mM L-glutamine, 10 μg/ml insulin and 50 μg/ml gentamycin. Cells were routinely cultured at 37°C with 5% CO_2_. All experiments were carried out using cells in the logarithmic growth phase.

### RNA interference by siRNA

Cells were trypsinized and plated at a density of 4 × 10^5 ^cells/cm^2 ^in six-well plates in fresh medium. After 24 hours the medium was replaced and cells were transfected with *Silencer *predesigned siRNAs (30 nM final concentration in culture medium; Ambion/Applied Biosystems, Foster City, CA, USA) using RNAiFect reagent (Qiagen, Crawley, West Sussex, UK), according to the supplied protocol. For *Fau *knockdown, two different siRNAs were employed, termed FAU1 and FAU2 (Ambion siRNA ID 46005 and ID 10907; target exons 2/3 and 3/4, respectively). The negative control siRNA was *Silencer *negative control #1 siRNA (Ambion code 4611). For *Fau *and *Bcl-G *combined knockdown, FAU2 siRNA and Bcl-G siRNA (Ambion siRNA ID 120721; targeting exons 2) were used, either alone or in combination. In these experiments, negative control siRNA was used at 30 nM (control for single knockdowns) and at 60 nM (control for combined knockdowns). In all experiments, cells were incubated with siRNAs for 120 hours. For evaluation of transfection efficiency, parallel transfections were conducted with Cy3-labelled siRNA prepared using the Silencer siRNA labelling kit (Ambion), according to the supplied protocol. The proportion of cells exhibiting fluorescence was determined by microscopy 24 hours post transfection. Transfection efficiencies were routinely 80% to 85%.

### UV irradiation and determination of cell viability and apoptosis

A hand-held UVG-54 lamp (UVP Ltd, Cambridge, UK) was used for irradiation. Radiation from the lamp was routinely measured using a Blak-Ray UV (shortwave) intensity meter (model J-225; UVP Ltd., Cambridge, UK). Trypsinized cells were resuspended at 10^5 ^cells/ml medium and were exposed to UVC light in plastic petri dishes with the lids removed for 20/30 seconds at a distance of 25 cm (40/60 J/m^2^) or were mock-irradiated. Immediately after UV exposure, cells were centrifuged and resuspended in the same volume of fresh medium.

For determination of their colony-forming ability (clonogenic assays), cells (20 μl UVC-irradiated; 5 μl mock-irradiated) were added to 1.5 ml maintenance medium supplemented with 10% (v/v) cell-conditioned medium (prepared from log phase cells) and plated in six-well plates. After 3 weeks of incubation, colonies were stained with crystal violet and were counted. Data are expressed as colonies per 100,000 cells plated.

For determination of short-term cell viability and apoptosis, cells were plated in 12-well plates (8 × 10^4 ^cells/well) in maintenance medium, incubated for 48 hours, and then trypsinized. Cell viability was determined by the nigrosin blue dye exclusion analysis. Apoptosis was determined by fluorescence microscopy; by assessment of either nuclear morphology or caspase activation. For the former assay, cells were stained with acridine orange (25 μg/ml), and the proportion of cells containing condensed or fragmented chromatin was scored. For the latter assay, the CaspaTag™ Fluorescein Caspase Activity Kit (Chemicon, Chandler's Ford, Hampshire, UK) was used, according to the manufacturer's instructions.

### Real-time RT-PCR

For cell culture samples, total RNA was isolated using TRIZOL reagent (Invitrogen, Paisley, UK). For clinical samples, paired tumour and adjacent normal breast epithelial tissues were collected from a total of 21 female patients with ductal breast cancer, rapidly frozen and stored at -140°C. All samples were examined histologically, and samples grossly contaminated with adipocytes, or with noncancerous tissue in the case of tumour samples, were excluded from the study. Total RNA was isolated using 1.4 M guanidine thiocyanate/0.5% sodium dodecyl sulphate/25 mM ethylenediamine tetraacetic acid/50 mM Tris–Cl (pH 7.5) [24,25]. For all samples, isolated RNA was treated with RQ1 RNase-free DNase (Promega, Southampton, Hampshire, UK), and was reverse transcribed using random hexamer priming and SuperScript II Reverse Transcriptase (Invitrogen), according to the supplied protocols. Real-time PCR was conducted using the Sensimix (dT) DNA kit (Quantace, Finchley, London, UK) and Taq Man Gene Expression Assays (assay codes Hs00609872_g1 for Fau, Hs00373302_m1 for Bcl-G, Hs00207681_m1 for MELK, Hs00167441_m1 for ALAS1, and Hs99999901_m1 for 18S; Applied Biosystems, Foster City, CA, USA), as recommended by the manufacturers, and was run on an ABI Prism Sequence Detection System model 7000 (Applied Biosystems, Foster City, CA, USA).

A standard curve, comprising cDNA prepared from the T-47D parental cell line, was included with each run to allow relative quantitation. Assays usually contained 0.1 to 30 ng standard (approximately threefold serial dilutions) or 5 ng sample cDNA in a final volume of 25 μl. For quantitation of Bcl-G, however, the sample and standard input were increased to 40 ng and 0.6 to 200 ng cDNA, respectively. For each assay, a standard curve of threshold cycle value versus log input standard cDNA was constructed by linear regression, and the equation of the line was used to calculate input amounts of samples from their respective threshold cycle values. Data were expressed relative to an endogenous control gene (Bcl-G sample values were first corrected for increased sample input). ALAS1 was used as the endogenous control gene, since the ALAS1/18S rRNA ratio is similar in breast ductal carcinoma and normal tissue, as described elsewhere [26].

### Statistical analysis

Data are presented as the mean and standard error of the mean, and statistical significance was determined either by a paired Student's *t *test or by one-way analysis of variance with Bonferroni's multiple comparison test for *post-hoc *analysis of selected groups, as specified in each case, depending upon the number of groups to be compared. Homogeneity of variance was checked by Bartlett's test and, where necessary, data were transformed (log or square root) prior to analysis.

### Correlation of gene expression with breast cancer patient survival

The analysis of gene expression using microarrays in a cohort of 99 breast carcinoma patients and the correlation of this with the survival data for these patients were as previously reported [27]. Total RNA was isolated from frozen tumours retrieved from Nottingham Hospitals NHS Trust Tumour Bank between 1986 and 1992 as described elsewhere [27]. RNA integrity and genomic DNA contamination were analysed using an Agilent 2100 Bioanalyzer (Agilent Technologies, Palo Alto, CA, USA). Total RNA was biotin-labelled using the Illumina TotalPrep RNA Amplification kit (Ambion) following the manufacturer's instructions. Biotin-labelled cRNA (1.5 μg) was used for each hybridization on Sentrix Human-6 BeadChips (Illumina, San Diego, CA, USA) in accordance with manufacturer's protocol. Illumina gene expression data containing 47,293 features were processed and summarized in the Illumina BeadStudio software. Analyses of the probe level data were performed using the *beadarray *Bioconductor package (Illumina Cambridge, Saffron Walden, Essex, UK). The expression data have been deposited in ArrayExpress at the European Bioinformatics Institute [EBI:E-TABM-576] [28].

Normalized expression of genes was dichotomized into low and high expression using the median value (Fau, range 10.05 to 11.60, median 10.96; MELK, range 5.63 to 8.25, median 6.20; Bcl-G, range 5.62 to 6.02, median 5.75).

## Results and discussion

**Figure 1:**
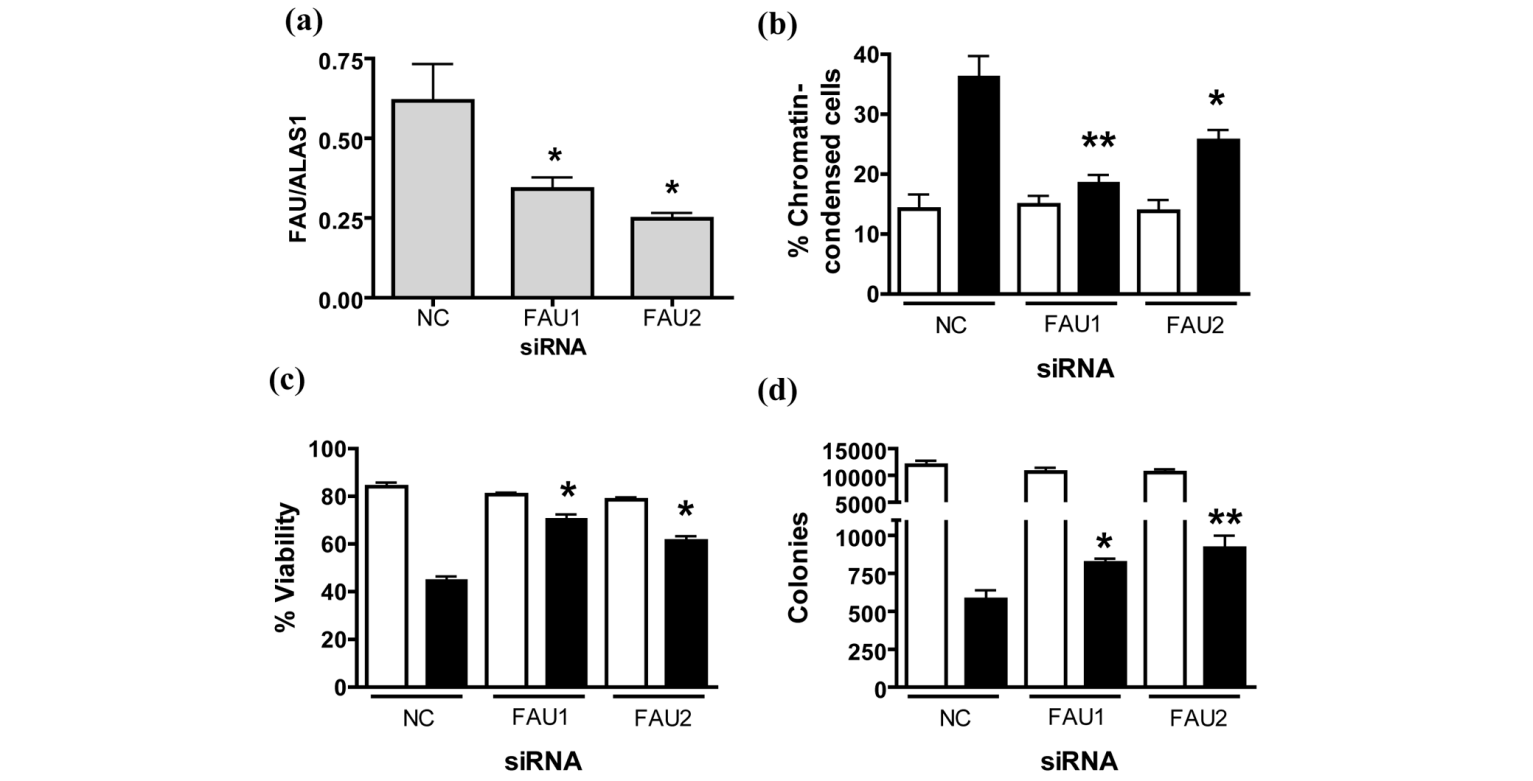
Downregulation of *Fau *inhibits UV-induced apoptosis of T-47D breast cancer cells. T-47D cells were transfected with one of two different siRNAs to *Fau *or with a negative control (NC) (Ambion code 4611) siRNA using RNAiFect (Qiagen). After 120 hours samples were collected for real-time RT-PCR analysis, and trypsinized cells were either exposed to UV light (40 J/m^2^; closed bars) or were mock-irradiated (open bars), and then replated in fresh medium. **(a) **Real-time RT-PCR analysis of *Fau *transcript levels. Data, expressed relative to the house-keeping gene *ALAS1*, are the mean ± standard error of the mean. *P < 0.05 versus NC siRNA (one-way analysis of variance (ANOVA) with Bonferroni's multiple comparison test); n = 3. **(b) **The proportion of apoptotic cells was determined 48 hours post UV exposure by acridine orange staining and fluorescence microscopy. *P < 0.05, **P < 0.01 versus NC siRNA (UV-irradiated; one-way ANOVA with Bonferroni's multiple comparison test); n = 3. **(c) **Short-term cell viability was determined 48 hours post UV exposure by dye exclusion. *P < 0.01 versus NC siRNA (UV-irradiated; one-way ANOVA with Bonferroni's multiple comparison test); n = 3. **(d) **Long-term cell viability after UV irradiation was determined in further cultures by measuring colony formation; colonies were counted 3 weeks post UV exposure. *P < 0.05, **P < 0.01 versus NC siRNA (UV-irradiated; one-way ANOVA with Bonferroni's multiple comparison test); n = 4.

The functional importance of endogenous *Fau *in the induction of apoptosis in breast cancer cells is indicated by the effects produced by reducing *Fau *expression in the T-47D breast cancer cell line using siRNAs. Two siRNAs termed FAU1 and FAU2 (target exons 2/3 and 3/4, respectively) were used, and each reduced endogenous *Fau *expression, as determined by real-time RT-PCR (Figure 1a). *Fau *knockdown markedly attenuated the UV-induced apoptosis of T-47D breast cancer cells (Figure 1b) and protected short-term cell viability (Figure 1c). Importantly, siRNA-mediated *Fau *silencing also increased the long-term survival of T-47D cells after UV irradiation, as measured using a colony-forming assay (Figure 1d). These effects are entirely consistent with the inhibition of T-cell apoptosis induced by downregulation of *Fau *with *Fau *antisense [15], and with the effects of *Fau *siRNAs on the embryonic kidney cell line HEK 293T and the prostate cell line 22Rv1 (Pickard MR and Williams GT, unpublished data), indicating the general validity of these observations.

**Figure 2:**
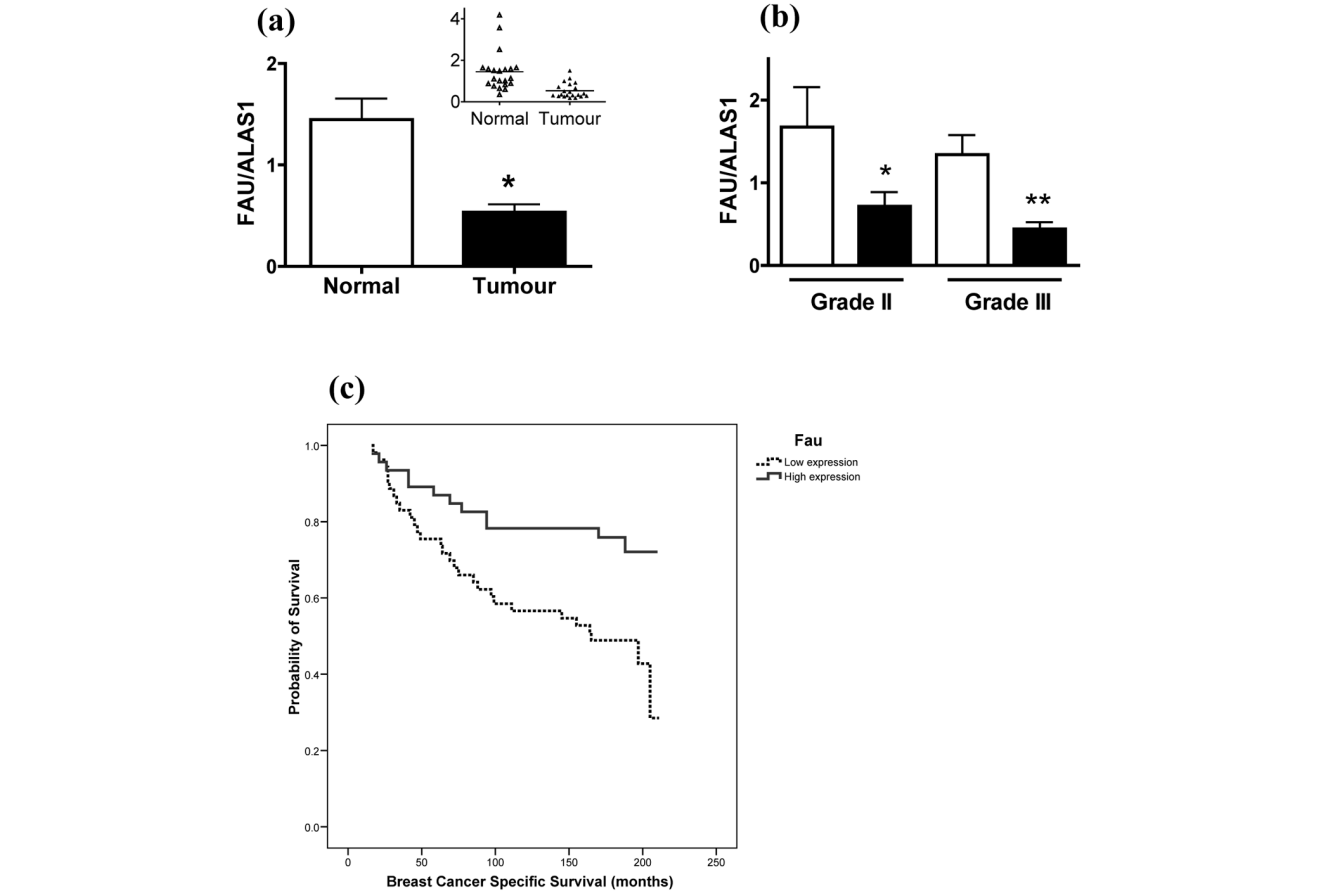
Reduced Fau transcript levels in breast cancer are associated with poor patient survival. **(a) **RNA was isolated from tumour tissue and adjacent normal tissue (n = 21 patients), was reverse transcribed, and *Fau *and *ALAS1 *transcript levels were determined using Taqman assays and real-time PCR using a relative standard curve protocol (cDNA from T-47D cells as standard). The *Fau/ALAS1 *transcript ratio is reduced in tumour tissue. *P = 0.002; paired Student's *t *test. Inset: scattergram of data. **(b) ***Fau *transcript levels are also reduced in tumour tissue (closed bars) versus normal tissue (open bars) in subsets of patients with grade II and grade III disease. *P < 0.05; n = 7 and **P < 0.001; n = 12; one-way analysis of variance with Bonferroni's multiple comparison test. **(c) **Kaplan–Meier survival curve showing reduced overall breast-cancer-specific survival in invasive breast cancer with lower Fau expression levels (*P *= 0.006).

Since serial analysis of gene expression has identified *Fau *as a downregulated transcript in ductal carcinoma *in situ *when compared with normal breast epithelium [18], we employed a real-time RT-PCR approach to determine *Fau *transcript levels in paired tumour and adjacent normal epithelial tissue from women with ductal carcinoma of the breast. The analysis shows a substantial and statistically significant reduction in *Fau *mRNA levels in breast ductal carcinoma samples (Figure 2a), both for patients with grade II disease (42% control value) and for patients with grade III disease (33% control value) (Figure 2b).

A further independent cohort of 99 primary operable invasive breast carcinomas presenting between 1986 and 1992 from the Nottingham-Tenovus Series, with long-term clinical follow-up, have been analysed previously using gene microarrays [27]. Normalized *Fau *expression was dichotomized using median levels and was associated with clinical outcome, revealing that lower gene expression of *Fau *was clearly correlated with a significant reduction in the breast-cancer-specific survival of patients (Figure 2c). The difference in survival of those with high expression versus low expression of *Fau *is both striking and statistically significant (*P *= 0.006), indicating that higher expression of Fau has a protective effect – as predicted for a candidate tumour suppressor.

**Figure 3:**
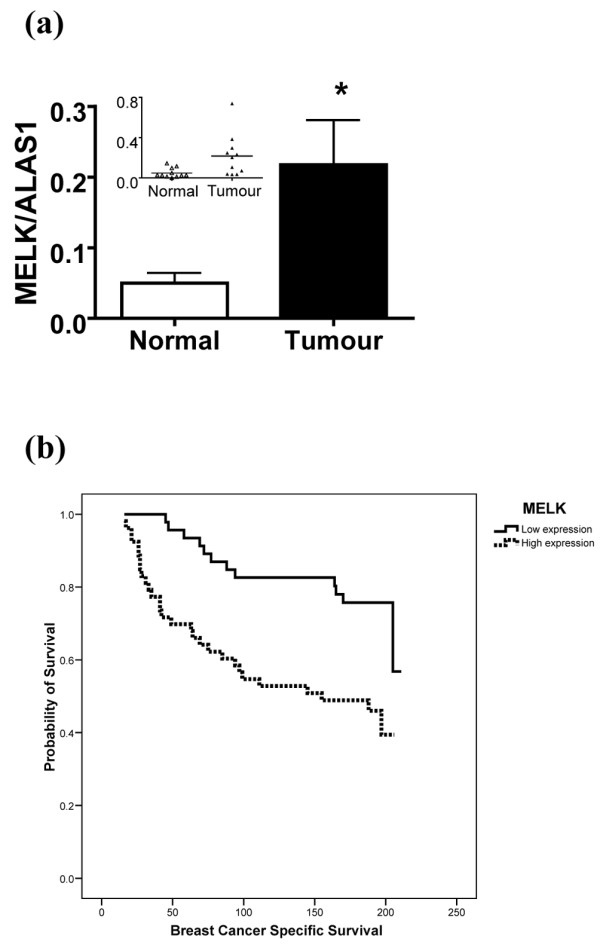
Increased MELK transcript levels in breast cancer are associated with poor patient survival. **(a) **RNA was isolated from tumour and adjacent normal tissue (n = 11 patients), was reverse transcribed, and *MELK *and *ALAS1 *transcript levels were determined using Taqman assays and real-time PCR using a relative standard curve protocol (cDNA from T-47D cells as standard). The *MELK/ALAS1 *transcript ratio is elevated in tumour tissue. *P = 0.022; paired Student's *t *test on log-transformed data. Inset: scattergram of data. **(b) **Kaplan–Meier survival curve showing significantly reduced overall breast-cancer-specific survival in invasive breast cancer with higher *MELK *expression levels (*P *= 0.001).

Novel protein kinase MELK has, like Fau, been shown to regulate Bcl-G [22]. We therefore analysed the level of *MELK *expression by real-time RT-PCR in matched breast cancer tissue and unaffected breast epithelial tissue from the same patients (Figure 3a). *MELK *expression is significantly upregulated in the breast cancer samples, confirming and extending the independent observations made by other laboratories on unmatched breast cancer tissue and normal tissue [22,23]. This increase in *MELK *expression in cancer, together with the observation that downregulation of *MELK *suppresses the growth of breast cancer cells *in vitro *[22], is fully consistent with the putative role of *MELK *as an oncogene. We therefore analysed the relationship between *MELK *expression levels and breast cancer patient survival in the same cohort of 99 patients used for the study on Fau expression (above). Higher *MELK *expression shows a strong correlation with poor survival in breast cancer patients (Figure 3b), supporting the suggestion that *MELK *expression is indeed an important factor in the clinical progression of breast cancer.

**Figure 4:**
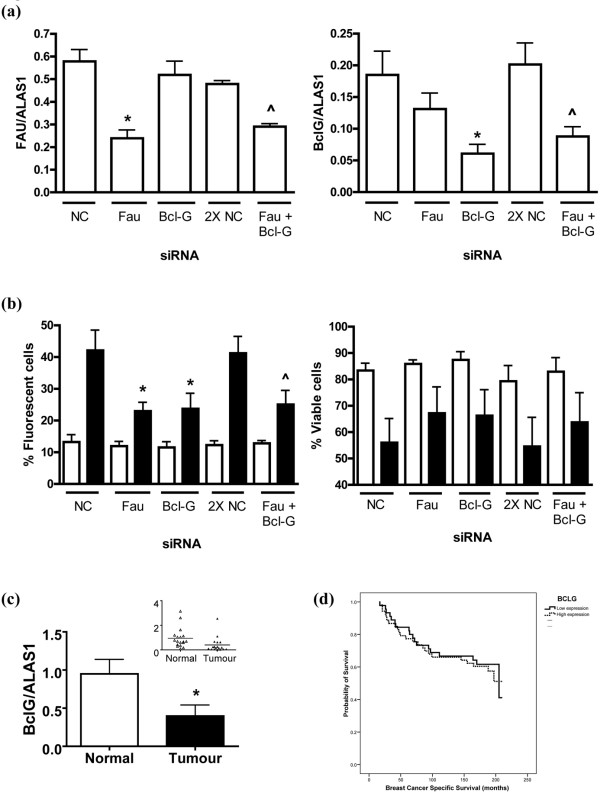
**siRNA-mediated knockdown of *Fau *and *Bcl-G *expression attenuates UV-induced apoptosis in breast cancer cells**. siRNA-mediated knockdown of *Fau *and *Bcl-G *expression, either alone or in combination, attenuates UV-induced apoptosis of T-47D breast cancer cells. T-47D cells were transfected with siRNA to *Fau *(FAU2), *Bcl-G *or negative control (NC). To observe the effects of combined knockdown, Fau plus Bcl-G siRNAs were co-transfected (Fau + Bcl-G); the controls for this group were cells transfected with double the amount of NC siRNA (2× NC). At 120 hours post transfection, samples were collected for the determination of *Fau *and *Bcl-G *transcript levels, and cells were either exposed to UV light (40 J/m^2^) or were mock-irradiated. **(a) **Real-time RT-PCR analysis of *Fau *(left-hand panel) and *Bcl-G *(right-hand panel) transcript levels. Data, expressed relative to the housekeeping gene *ALAS1*, are the mean ± standard error of the mean. *P < 0.05 versus NC siRNA, ^*P *< 0.05 versus 2× NC siRNA (Bonferroni's multiple comparison test); n = 5. **(b) **At 48 hours post UV exposure, the proportion of apoptotic cells (left-hand panel) was determined by a CaspaTag assay and fluorescence microscopy, and the short-term cell viability (right-hand panel) was determined by dye exclusion. Data for mock-irradiated (light bars) and UV-treated (dark bars) cells are the mean ± standard error of the mean. *P < 0.05 versus NC siRNA (UV-irradiated), ^*P *< 0.05 versus 2× NC siRNA (UV-irradiated) (Bonferroni's multiple comparison test); n = 5. Note that knockdown of either Fau or Bcl-G alone or in combination attenuates apoptosis induction by UV and that the extent of inhibition is similar for all three treatments. *(c) Bcl-G *transcript levels are reduced in ductal carcinoma of the breast. RNA was isolated from tumour and adjacent normal tissue (n = 18 patients), was reverse transcribed, and *Bcl-G *and *ALAS1 *transcript levels were determined using Taqman assays and real-time PCR using a relative standard curve protocol (cDNA from T-47D cells as standard). The *Bcl-G/ALAS1 *transcript ratio is reduced in tumour tissue. *P = 0.0021; paired Student's *t *test. Inset: scattergram of data.**(d) **Expression of Bcl-G was analysed in a cohort of 99 breast carcinoma patients and was correlated with survival data, as previously reported [27]. Kaplan–Meier survival curve showing no significant correlation between total *Bcl-G *expression levels and patient survival.

MELK has already been shown to modulate Bcl-G activity in breast cancer cells [22], but Fau has previously been shown to modulate Bcl-G only in mammalian leukocyte cell lines [21] (Pickard MR and Williams GT). We therefore used RNA interference to downregulate both *Fau *and *Bcl-G *in breast cancer cells, in order to determine whether they act in the same pathway in these cells. Downregulation of either *Fau *or *Bcl-G *independently had a significant inhibitory effect on UV-induced apoptosis, confirming the importance of both of these molecules in the induction of apoptosis (Figure 4). This effect was clearly demonstrated both by reductions in the proportion of cells staining for active caspases and by increases in the proportion of viable cells (Figure 4b). Both graphs also show that the simultaneous downregulation of both Bcl-G and Fau does not produce any additive protection. This is again consistent with both of these genes acting in the same pathway; that is, with the apoptosis-controlling effects of Fau being mediated by Bcl-G.

The striking correlations between changes in *Fau *and *MELK *expression and breast cancer progression focused attention on their common target, Bcl-G, in order to determine whether *Bcl-G *expression levels, or the post-translational modifications by Fau and MELK, were more important in controlling its activity. Real-time RT-PCR analysis of *Bcl-G *expression in breast cancer samples and matched normal samples indicated that *Bcl-G *expression was indeed reduced in breast cancer samples (Figure 4c). Analysis of the relationship between *Bcl-G *expression levels and breast cancer patient survival in the cohort of breast cancer patients examined for Fau and MELK (above), however, did not indicate any significant correlation (Figure 4d). This suggests that the regulation of Bcl-G activity by post-translational modification is more important than the *Bcl-G *expression level itself in determining breast cancer patient survival.

A notable combination of clinical studies and observations on breast cancer cells *in vitro *converge on the functionally connected Fau, Bcl-G and MELK molecules, and indicate that they are of considerable importance in breast cancer progression. This makes them attractive candidates for use in prognosis, risk prediction and targeted prevention, as for other breast cancer susceptibility genes [29]. *In vitro *studies from our own laboratory (Figures 1 and 4 above) and other laboratories [22] demonstrate that changes in the expression levels of these genes affect breast cancer cell susceptibility to apoptosis, indicating that the cancer-associated changes in gene expression observed are of functional as well as diagnostic importance, and suggesting several targets for cancer therapies. As a protein kinase, MELK is a particularly suitable target for drug therapy, given the mounting successes from specifically inhibiting other kinases in cancer therapy [30,31]. In addition, the interaction of BH3-only Bcl-2-related proteins such as Bcl-G with other members of the Bcl-2 family is a crucial stage in the induction of apoptosis and can be mimicked with small molecular weight candidate drug molecules [32,33], emphasizing the clinical importance of the investigation of these interactions for breast cancer treatment.

## Conclusions

The functionally inter-connected proteins Fau, Bcl-G and MELK play critical roles in the control of apoptosis that are central to breast cancer development and therapy. Reduced expression of candidate tumour suppressor *Fau *in breast cancer cells is associated with poor patient survival. Increased expression of candidate oncogene *MELK *is also associated with poor prognosis. Both Fau and MELK act, at least in part, through covalent modification of apoptosis controller Bcl-G.

## Abbreviations

Fau: Finkel–Biskis–Reilly murine sarcoma virus-associated ubiquitously expressed gene; FCS: foetal calf serum; FUBI: Fau ubiquitin-like domain; MELK: maternal embryonic leucine zipper kinase; MEM: minimal essential Eagle's medium; PCR: polymerase chain reaction; RT: reverse transcriptase; siRNA: small interfering RNA.

## Competing interests

The authors declare that they have no competing interests.

## Authors' contributions

MRP performed the *in vitro *studies and statistical analyses, and drafted the manuscript. ARG analysed the microarray gene expression and breast cancer patient survival data. IOE and CC both provided microarray gene expression and survival data. VLH analysed gene expression in the matched cancer and normal tissue samples. MM-M performed the initial studies on Fau. GTW designed the study and revised the manuscript. All authors read and approved the final manuscript.
